# The frontotemporal syndrome of ALS is associated with poor survival

**DOI:** 10.1007/s00415-016-8290-1

**Published:** 2016-09-26

**Authors:** Rosanne Govaarts, Emma Beeldman, Mike J. Kampelmacher, Marie-Jose van Tol, Leonard H. van den Berg, Anneke J. van der Kooi, Peter J. Wijkstra, Marianne Zijnen-Suyker, Nicolle A. M. Cobben, Ben A. Schmand, Rob J. de Haan, Marianne de Visser, Joost Raaphorst

**Affiliations:** 1 Department of Neurology, Academic Medical Center, University of Amsterdam, Amsterdam, The Netherlands; 2 Center for Home Mechanical Ventilation, University Medical Center Utrecht, Utrecht, The Netherlands; 3 Neuroimaging Center, University Medical Center Groningen, University of Groningen, Groningen, The Netherlands; 4 Department of Neurology, Brain Center Rudolf Magnus Institute of Neuroscience, University Medical Center Utrecht, Utrecht, The Netherlands; 5 Department of Home Mechanical Ventilation/Pulmonary Diseases, University Medical Center Groningen, University of Groningen, Groningen, The Netherlands; 6 Department of Intensive Care/Center for Home Mechanical Ventilation, University Medical Center, Erasmus MC, Rotterdam, The Netherlands; 7 Department of Respiratory Medicine/Center of Home Mechanical Ventilation Maastricht, Maastricht University Medical Center, Maastricht, The Netherlands; 8 Department of Medical Psychology, University of Amsterdam, Amsterdam, The Netherlands; 9 Clinical Research Unit, Academic Medical Center, University of Amsterdam, Amsterdam, The Netherlands; 10 Department of Neurology, Radboud University Medical Center Nijmegen, Nijmegen, The Netherlands

**Keywords:** Amyotrophic lateral sclerosis, Frontotemporal syndrome, Cognitive impairment, Behavioral changes, Survival, Non-invasive ventilation

## Abstract

Thirty percent of ALS patients have a frontotemporal syndrome (FS), defined as behavioral changes or cognitive impairment. Despite previous studies, there are no firm conclusions on the effect of the FS on survival and the use of non-invasive ventilation (NIV) in ALS. We examined the effect of the FS on survival and the start and duration of NIV in ALS. Behavioral changes were defined as >22 points on the ALS-Frontotemporal-Dementia-Questionnaire or ≥3 points on ≥2 items of the Neuropsychiatric Inventory. Cognitive impairment was defined as below the fifth percentile on ≥2 tests of executive function, memory or language. Classic ALS was defined as ALS without the frontotemporal syndrome. We performed survival analyses from symptom onset and time from NIV initiation, respectively, to death. The impact of the explanatory variables on survival and NIV initiation were examined using Cox proportional hazards models. We included 110 ALS patients (76 men) with a mean age of 62 years. Median survival time was 4.3 years (95 % CI 3.53–5.13). Forty-seven patients (43 %) had an FS. Factors associated with shorter survival were FS, bulbar onset, older age at onset, short time to diagnosis and a C9orf72 repeat expansion. The adjusted hazard ratio (HR) for the FS was 2.29 (95 % CI 1.44–3.65, *p* < 0.001) in a multivariate model. Patients with an FS had a shorter survival after NIV initiation (adjusted HR 2.70, 95 % CI 1.04–4.67, *p* = 0.04). In conclusion, there is an association between the frontotemporal syndrome and poor survival in ALS, which remains present after initiation of NIV.

## Introduction

Thirty to 50 percent of amyotrophic lateral sclerosis (ALS) patients have a frontotemporal syndrome, encompassing behavioral changes and cognitive impairment [17, 20, 24, 27, 38]. In 8–10 % of ALS patients these changes are more severe and fulfill the criteria for frontotemporal dementia (FTD) [24, 28]. The most frequent encountered subtype of FTD is the behavioral variant (bvFTD) albeit language variants of FTD may be found in a minority of ALS patients. Median survival of ALS patients is 3 years after symptom onset and the main cause of death is respiratory failure. The disease course is negatively influenced by older age at onset, early respiratory dysfunction, bulbar onset, a short time to diagnosis and the presence of a C9orf72 repeat expansion [6, 11, 12, 15, 34]. Previous studies suggested a negative effect of executive dysfunction or neurobehavioral changes on survival of ALS patients, although the use of general measures for behavioral changes precluded firm conclusions in some of them [10, 15, 18, 23, 26]. Indeed, the importance of a valid assessment of the frontotemporal syndrome in ALS, including the use of disease-specific measures has been stressed [17]. This would allow for substantiating the presumed association between the frontotemporal syndrome and survival in ALS [10, 15, 26]. The frontotemporal syndrome, in particular the presence of behavioral changes, was found to interfere with the initiation of life-prolonging therapies, i.e., non-invasive ventilation (NIV). An analysis of both cognitive and behavioral changes in patients who used NIV might corroborate this association [10, 22, 26].

The first aim of this study was to investigate whether a frontotemporal syndrome is an independent risk factor for poor survival in ALS. Second, we aimed to gain insight into the effect of the frontotemporal syndrome on NIV initiation and duration in ALS patients.

## Methods

### Participants

In two of our previous studies, behavioral changes and cognitive impairment were assessed in a total of 110 ALS patients (*n* = 21 and *n* = 89, respectively) [29, 30]. ALS patients in both studies were diagnosed with possible, probable or definite ALS according to the El Escorial criteria [5]. Concomitant FTD was diagnosed by the treating neurologist, according to the Neary criteria [25]. Both patient cohorts were recruited from the two tertiary referral clinics for ALS in the Netherlands. Demographic and clinical data were extracted from the databases, i.e., age, years of education, site of onset (bulbar or limb), age at onset, time to diagnosis, disease duration (time between first symptom and study visit), vital capacity at inclusion (percentage of the predicted value, as measured by handheld spirometry in the upright position), and score on the Hospital Anxiety and Depression scale (HADS; a maximum score of 42 indicates severe anxiety and depression) [39]. Physical disability was measured with the amyotrophic lateral sclerosis functional rating scale-revised (ALSFRS-R; maximum score of 48 indicates no physical disability) [8].

Data on the presence of the C9orf72 repeat expansion were derived from the prospective population-based study on motor neuron disease in the Netherlands [19]. DNA was extracted from venous blood using standard protocols [37]. To detect large expanded repeats, a repeat primed PCR for the C9ORF72 GGGGCC repeat was performed on genomic DNA, as described previously [37]. The C9orf72 repeat expansion was known in 89 (81 %) patients in the current study. Time of death was checked in the Municipal Personal Records Database [14].

### Standard protocol approvals and patient consents

The medical ethical committees of the hospitals approved the studies. Written informed consent was obtained from all participants at inclusion.

### Assessment of behavior and cognition

In the first cohort (*n* = 21), behavioral changes were assessed with the Neuropsychiatric Inventory (NPI) [13, 30]. A score of ≥3 points on ≥2 items on the NPI has been used by others as a cutoff for mild behavioral changes in ALS [29, 35]. As described previously by others, we carefully excluded behavioral changes that are more likely due to physical disability (i.e., motor impairment mistaken for apathy) or an appropriate reaction to their diagnosis (e.g., comments about death). Cognitive functions were assessed with a comprehensive neuropsychological examination. Neuropsychological tests were corrected for age, education and dysarthria, as described previously [2, 30]. Cognitive impairment was defined as a score below the fifth percentile on ≥2 tests of executive function, memory or language, according to consensus criteria for cognitive impairment in ALS [35].

In the second cohort (*n* = 89), behavioral changes were assessed with the ALS-FTD-Questionnaire (ALS-FTD-Q). The ALS-FTD-Q is a proxy-rated, disease-specific, validated instrument to detect behavioral changes in ALS [29]. The items of the questionnaire are based on a systematic review of behavioral changes in motor neuron disease patients with the behavioral variant of FTD [28]. A score of >22 on the ALS-FTD-Q indicates mild behavioral changes [29]. Cognitive functions in the second cohort were assessed with the category fluency test, letter fluency test and the Mini Mental State Examination (MMSE). Cognitive impairment was defined as ≥2 impaired tests [35]. For the fluency tests we used a cutoff below the fifth percentile of normative scores [33]. For the MMSE, an age and education normative score was used which was adjusted for the highest achievable score of individual patients; some patients could not complete all items due to motor impairment [16].

### Classification of patients

Based on consensus criteria for the frontotemporal syndrome in ALS, we defined the following groups: classic ALS, which is not associated with cognitive impairment or behavioral changes, and ALS patients with a frontotemporal syndrome [35]. The frontotemporal syndrome was defined as cognitive impairment, behavioral changes or both. Patients with severe cognitive and behavioral changes who fulfilled the criteria for the behavioral variant of FTD were included in the group “ALS patients with a frontotemporal syndrome” [32].

### Non-invasive ventilation

There are four Home Mechanical Ventilation centers in the Netherlands from which data were derived on whether the patient started NIV, and the dates on which the NIV was initiated and ended. We also noted whether and when tracheostomal ventilation was initiated.

### Statistical analyses

Survival was calculated as time from symptom onset to time of death or censoring date (February 24, 2015). As no patients had tracheostomal ventilation, survival following onset of NIV was calculated as time from onset of NIV to time of death or censoring date (July 2014). We used Kaplan–Meier survival analyses and compared the data using the log-rank test. To examine which explanatory variables should be included in the multivariate Cox proportional hazards model, we performed univariate analyses for the following factors: frontotemporal syndrome, bulbar onset, age at onset, time to diagnosis, vital capacity, gender and the presence of the C9orf72 repeat expansion. Explanatory variables with a *p* value <0.25 in the univariate analyses were included into a multivariate Cox proportional hazards model (using the enter method). Effect sizes were expressed in hazard ratios, statistical uncertainty was expressed in 95 % confidence intervals. The defined groups, classic ALS and ALS patients with a frontotemporal syndrome, were compared using independent *t* test, Pearson Chi-squared test and Fisher’s exact test. All tests were two-tailed and statistical significance was set at *p* < 0.05. Statistical analyses were carried out using SPSS version 21 (SPSS Inc., Chicago, IL).

## Results

**Fig. 1:**
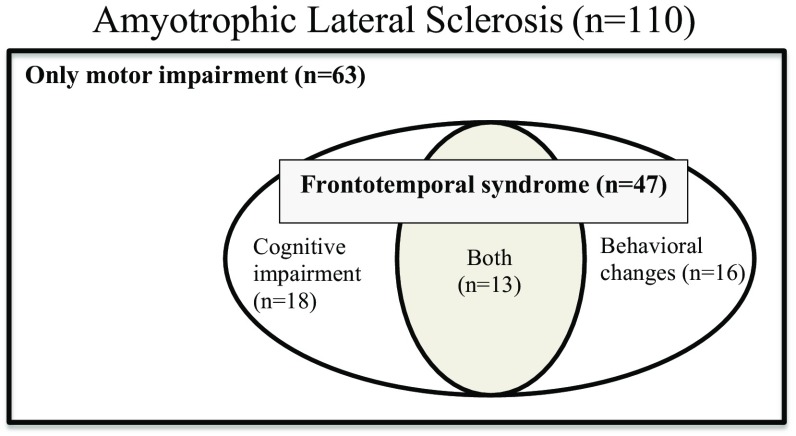
Venn diagram of the classification of amyotrophic lateral sclerosis patients (*n* = 110)

In total 110 ALS patients were included; 76 men and 34 women; 22 (20 %) ALS patients had bulbar onset. The mean age was 62.0 years (SD 11.3; range 34–84). Median time from symptom onset to diagnosis was 10 months (range 2–90). The cognitive assessment was done at a median disease duration after symptom onset of 3.3 years (range 0.6–27.3). Forty-seven (43 %) ALS patients had a frontotemporal syndrome, of whom 8 fulfilled criteria for ALS-bvFTD. Sixteen (15 %) patients had only behavioral changes, 18 (16 %) had cognitive impairment and 13 (12 %) had both (Fig. 1). None of the patients had a language variant of FTD. There was no difference in occurrence of the frontotemporal syndrome between the two cohorts (χ^2^, *p* = 0.99) [29, 30]. Demographic and clinical variables for the patient groups are shown in Table 1.

**Table 1:** Demographic and clinical characteristics of ALS patients

	ALS-classic (*n* = 63)	ALS-frontotemporal syndrome (*n* = 47)	ALS-frontotemporal syndrome (*n* = 47)		
			ALS-cognitive impairment (*n* = 18)	ALS-behavioral changes (*n* = 16)	ALS-both (*n* = 13)
Age at onset, years	56.5 (12.8)	61.6 (9.5)*	60.4 (9.1)	62.2 (8.9)	62.4 (11.2)
Male sex, *n* (%)	39 (61.9)	37 (78.7)	13 (72.2)	14 (87.5)	10 (76.9)
Bulbar onset, *n* (%)	6 (9.5)	16 (34.0)*	6 (33.3)*	4 (25.0)	6 (46.2)*
Time to diagnosis, months (range)	13.6 (1–71)	14.4 (1–76)	14.2 (1–36)	10.9 (1–47)	19.1 (1–76)
ALSFRS-R	34.2 (8.8)	31.5 (8.3)	28.4 (9.4)*	33.4 (7.1)	33.4 (7.3)
Vital capacity (%)	84.9 (22.4)	77.9 (17.1)	75.8 (21.3)	82.6 (12.9)	74.9 (14.8)
C9orf72 repeat expansion, *n* (%)	2 (3.2)	4 (8.5)	1 (5.6)	2 (12.5)	1 (7.7)
HADS-anxiety	10.2 (5.2)	10.5 (4.7)	10.9 (4.0)	9.1 (4.6)	11.8 (5.5)
HADS-depression	7.2 (3.7)	8.2 (3.7)	7.4 (2.4)	8.3 (4.1)	9.4 (4.6)
Education, years	13.9 (2.6)	14.1 (2.4)	14.6 (2.2)	13.3 (2.9)	14.4 (1.9)
NIV initiation, *n* (%)	29 (46.0)	14 (29.8)	8 (44.4)	2 (12.5)*	4 (30.8)

Values are mean (SD), unless stated otherwise. All groups are compared to the classic ALS group using an independent *t* test

*ALS* amyotrophic lateral sclerosis, *ALSFRS-R* ALS functional rating scale-revised (maximum score 48, indicates no disability), *HADS* Hospital anxiety and depression scale, *NIV* non-invasive ventilation, *y* years

* *p* < 0.05

### Survival analyses

At the time of analyses, 87 (79.1 %) patients had died. The overall median survival time from symptom onset to death was 4.3 years [95 % confidence interval (CI) 3.53–5.13]. Table 2 shows univariate analyses of risk factors for survival in all ALS patients. Frontotemporal syndrome, bulbar onset, age at onset, time to diagnosis and the presence of the C9orf72 repeat expansion had *p* values below 0.25 and were included in the multivariate model.

**Table 2:** Univariate analysis of possible risk factors in all ALS patients

Risk factor	Hazard ratio	95 % CI	*p* value
Bulbar onset	2.70	1.63–4.49	<0.001*
Age at onset	1.05	1.02–1.07	<0.001*
Time to diagnosis	0.97	0.95–0.99	0.001*
Vital capacity	1.00	0.99–1.01	0.544
Familial/sporadic ALS	1.25	0.64–2.42	0.515
Gender	1.23	0.77–1.96	0.385
C9orf72 repeat expansion	1.31	1.01–1.069	0.04*
Frontotemporal syndrome	2.09	1.36–3.21	0.001*
Behavioral changes	1.95	1.08–3.55	0.028*
Cognitive impairment	2.01	1.15–3.53	0.014*
Both	2.28	1.19–4.36	0.013*

*ALS* amyotrophic lateral sclerosis, *CI* confidence interval

* *p* < 0.25; these variables were included into a multivariate Cox proportional hazards model

### Survival of ALS patients with a frontotemporal syndrome

**Fig. 2:**
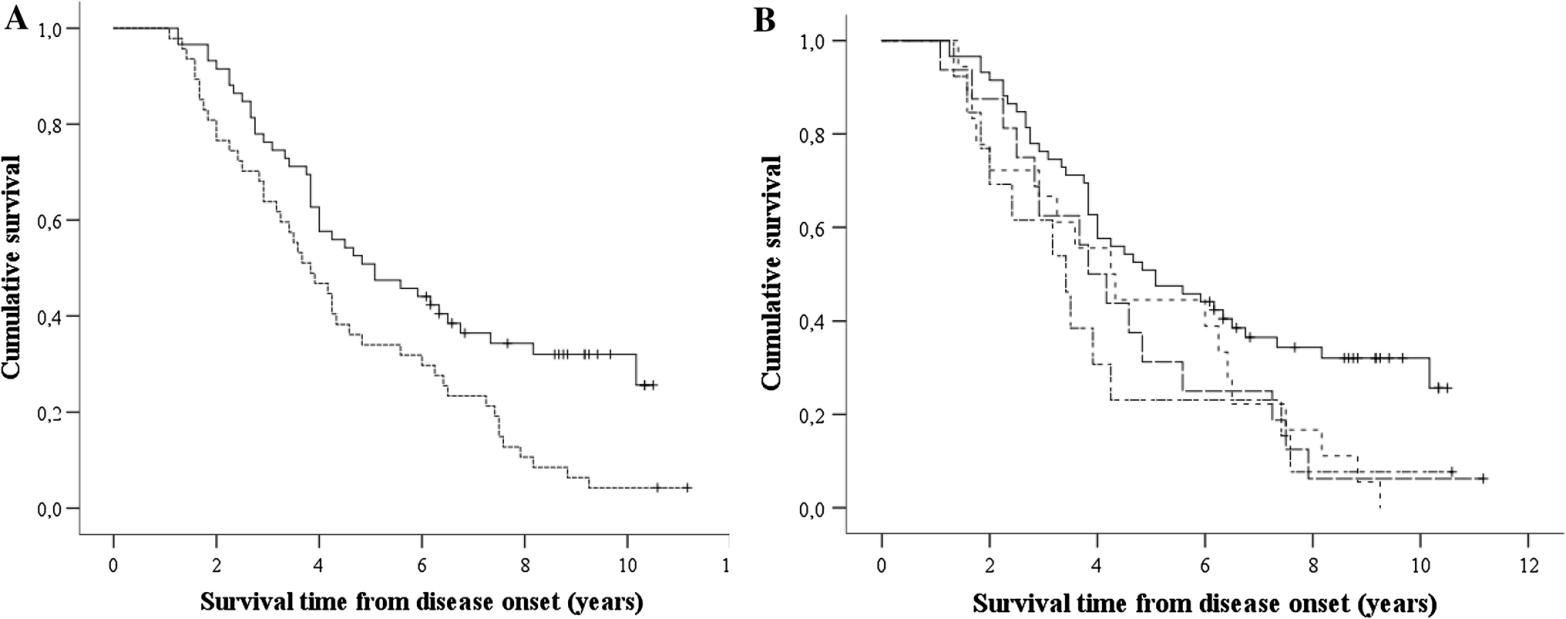
**a** Kaplan–Meier analysis of the effect of frontotemporal syndrome on ALS survival. Log-rank test for equality of survival functions, *p* = 0.005. *Black line* ALS patients without frontotemporal syndrome (*n* = 63); *dotted line* ALS with frontotemporal syndrome (*n* = 47); +: censored cases (*n* = 23). **b** Kaplan–Meier analysis of the effect of cognitive impairment and/or behavioral changes on ALS survival. *Black line* classic ALS patients, without frontotemporal syndrome (*n* = 63); *dotted line* ALS with cognitive impairment (*n* = 18, *p* = 0.012); *dashed line* ALS with behavioral changes (*n* = 16, *p* = 0.02); *dotted/dashed line* ALS with both cognitive impairment and behavioral changes (*n* = 13, *p* = 0.10); +: censored cases (*n* = 23); four patients (two censored) who had a survival time of 15.6–29 years are not displayed in this figure

Median survival time was shorter in 47 patients with a frontotemporal syndrome compared to 63 classic ALS patients (3.8 years, 95 % CI 2.94–4.73 vs. 5.6 years, 95 % CI 3.76–7.41, *p* = 0.001). The Kaplan–Meier curve is shown in Fig. 2a. The impact effect of a frontotemporal syndrome on survival was also observed in a multivariate regression model adjusting for bulbar onset, age at onset, time to diagnosis and the presence of the C9orf72 repeat expansion (adjusted hazard ratio 2.29, 95 % CI 1.44–3.65, *p* < 0.001; Table 3).

**Table 3:** Multivariate analysis for ALS patients with frontotemporal syndrome on survival

Risk factor	Hazard ratio	95 % CI	*p* value
Age of onset	1.04	1.02–1.06	0.001
Bulbar onset	1.93	1.15–3.24	0.013
Extended C9orf72 repeat	1.54	1.17–2.03	0.002
Time to diagnosis	0.96	0.93–0.98	<0.001
Frontotemporal syndrome	2.29	1.44–3.65	<0.001

*CI* confidence interval

### Survival of ALS patients with behavioral changes and/or cognitive impairment

ALS patients with both behavioral changes and cognitive impairment had a median survival time of 3.4 years (*n* = 13; 95 % CI 2.14–4.69, *p* = 0.01; Fig. 2b), compared to 5.6 years in classic ALS. ALS patients with cognitive impairment had a median survival time of 4.3 years (*n* = 18; 4.3 years, 95 % CI 2.69–5.81, *p* = 0.012; Fig. 2b). ALS patients with behavioral changes had a median survival time of 3.8 years (*n* = 16; 95 % CI 2.94–4.81, *p* = 0.024; Fig. 2b). Multivariate analysis for these subgroups was not performed, due to a small size.

### Non-invasive ventilation

Forty-three patients (39 %) had used NIV of whom 29 (67 %) had classic ALS and 14 (33 %) had ALS with a frontotemporal syndrome (*χ* ^2^, *p* = 0.08). Out of the 43 patients who had used ventilation, 8 (19 %) had bulbar onset. All except two patients had used NIV until death. Two patients used NIV 2 and 5 days and lived 11 and 18 days, respectively, following the termination of NIV. None of the patients had tracheostomal ventilation.

### Initiation of non-invasive ventilation

The median disease duration at NIV initiation was 3.9 years (range 0.5–8.2) for classic ALS and 3.1 years (range 1.0–7.0) for ALS patients with a frontotemporal syndrome (*p* > 0.05). Patients with behavioral changes initiated NIV less often compared to classic ALS patients (2 out of 16; 12.5 % vs. 29 out of 63; 46 %, Fisher exact *p* = 0.02). The proportion of ALS patients with cognitive changes (with or without behavioral changes) that initiated NIV did not differ from those with classic ALS.

### Survival after non-invasive ventilation

ALS patients who had used NIV had a longer survival than patients without NIV (median 6.0 years, 95 % CI 4.4–7.6 vs. 3.8 years 95 % CI 3.2–4.3, *p* = 0.03).

**Fig. 3:**
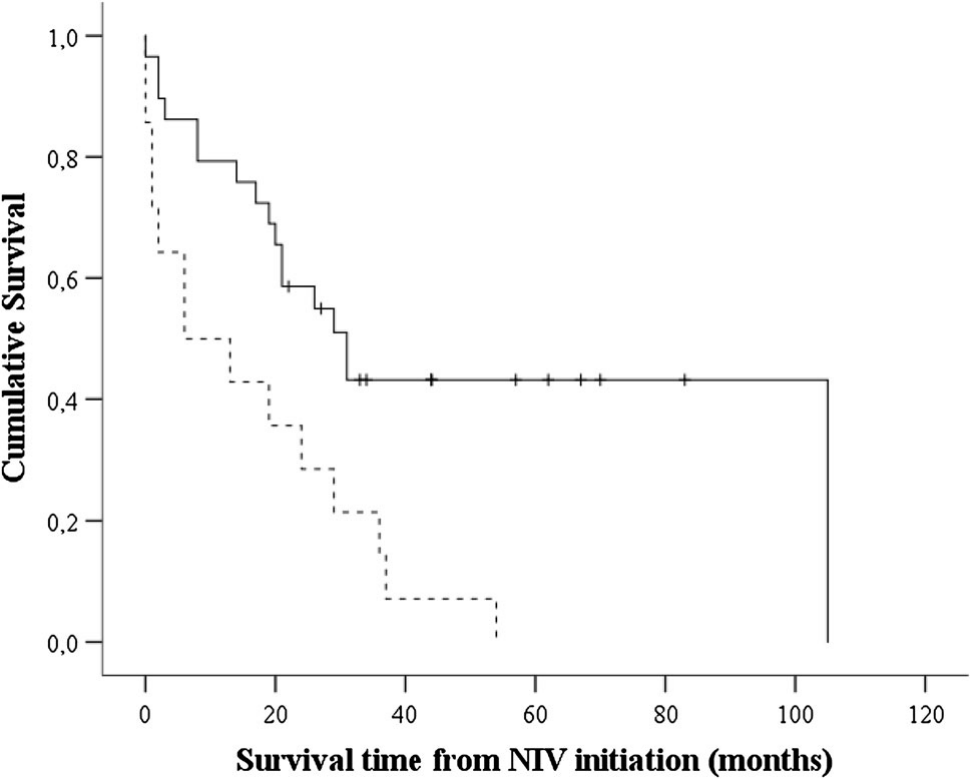
Kaplan–Meier analysis of the effect of the frontotemporal syndrome on ALS survival following initiation of NIV. Log-rank test for equality of survival functions, *p* = 0.003. *Black line* ALS patients without frontotemporal syndrome (*n* = 29); *dotted line* ALS with frontotemporal syndrome (*n* = 14); +: censored cases (*n* = 12)

ALS patients with a frontotemporal syndrome had a shorter survival after NIV initiation compared to those with classic ALS (median 6 months, 95 % CI 0.00–18.9 vs. 31 months 95 % CI 19.9–43.0, *p* = 0.009; Fig. 3). The impact effect of a frontotemporal syndrome on survival (following NIV) was also observed in a multivariate regression model adjusting for site of onset, age at onset, time to diagnosis and the presence of the C9orf72 repeat expansion (HR 2.7, 95 % CI 1.2–6.0, *p* = 0.02).

In an exploratory analysis, we analyzed the effect of the frontotemporal syndrome on survival in the non-ventilated group. Non-ventilated ALS patients with a frontotemporal syndrome showed a trend to a shorter survival compared to classic ALS patients without NIV (median 4.00 years, 95 % CI 3.43–3.82 vs. 3.59 years 95 % CI 2.55–4.62, *p* = 0.06).

## Discussion

In 110 patients with ALS, we examined the effect of the presence of the frontotemporal syndrome on survival and NIV initiation and duration. We showed that the concomitant presence of the frontotemporal syndrome (behavioral changes, cognitive impairment or both) in patients with ALS is associated with a significantly shorter survival independent of other prognostic factors. We also found that the survival after NIV initiation was significantly shorter in ALS patients with a frontotemporal syndrome compared to classic ALS. Thus, our findings suggest an association between the frontotemporal syndrome and poor survival in ALS patients, which remains present following the initiation of NIV.

Known factors for a poor prognosis are older age at onset, early respiratory dysfunction, bulbar onset, short time to diagnosis and the presence of the C9orf72 repeat expansion [6, 11, 12, 15, 34]. Early respiratory dysfunction was not a relevant prognostic factor in our study, possibly because the median vital capacity (percentage of predicted value) of our patients was 84 % at inclusion [30].

Our study strengthens and extends findings from previous studies as our data robustly showed that not only cognitive changes, but also behavioral changes negatively affect survival in ALS [10, 15, 18, 26]. Importantly, for the assessment of behavioral changes we used a disease-specific instrument which has shown to prevent overestimation of behavioral changes in ALS [29].

NIV is a life-prolonging therapy in ALS and, therefore, an important variable to consider in survival studies in ALS [4]. Reports on analyses of NIV use provide clues on possible causes of reduced survival in ALS patients with a frontotemporal syndrome [15, 26]. We showed that in particular ALS patients with behavioral changes (and not cognitive changes) less often initiated NIV, and that ALS patients with a frontotemporal syndrome had a shorter survival after NIV initiation compared to classic ALS patients. Our data, due to relatively low numbers, do not enable us to differentiate whether apathy, disinhibition or dysexecutive behavior mediates this difference. An association between frontotemporal dysfunction and either the initiation of NIV, or survival following NIV, has been shown by others [10, 26]. Our study extends these findings and suggests that a shorter survival due to a frontotemporal syndrome can be explained by both a lower proportion of ALS patients (with a frontotemporal syndrome) initiating NIV, and a shorter survival of ALS patients with a frontotemporal syndrome, following NIV [10, 26]. Larger cohorts are needed to confirm this association and other issues. In particular, more data should be obtained on compliance with NIV, the role of patients, proxies and physicians in the decision-making process, and on end of life practices in ALS patients with the frontotemporal syndrome [9, 21, 31]. In addition, a study in a larger cohort may elucidate the impact of different aspects of behavioral changes in ALS (i.e., apathetic vs disinhibited type) [7].

In addition to the strengths of our study, some limitations need to be addressed. The assessment of behavioral changes and cognitive impairment differed slightly between the two cohorts. However, no differences in outcomes were shown between the cohorts. We included more prevalent than incident patients, i.e., the disease duration was more than 1 year in most patients. This is reflected by a relatively long survival of the cohort, probably due to a lower proportion of patients with a rapid disease course. The cognitive testing in one cohort focused on executive functions, which may have resulted in an underestimation of cognitive deficits, because other cognitive domains can be affected in ALS, even in the absence of executive deficits. As fluency is an important aspect of executive functioning, the association of cognitive dysfunction with survival in our study may have in part be driven by executive dysfunction, thus corroborating previous findings from Elamin et al. [1, 3, 15, 36]. Due to small numbers we were not able to perform multivariate analysis in the subgroups (i.e., patients with only cognitive impairment, patients with only behavioral changes, and patients with both). Finally, we were unable to retrieve sufficient data on the initiation and duration of feeding by gastrostomy.

In conclusion, we have shown an effect of the frontotemporal syndrome on survival in ALS, which is in part related to a shorter NIV use as compared to classic ALS patients. These findings underline the importance of the assessment of cognitive impairment and behavioral changes in ALS patients and contribute to a better understanding of prognostic factors in ALS.
